# Lessons Learned from Public Health and State Prison Collaborations during COVID-19 Pandemic and Multifacility Tuberculosis Outbreak, Washington, USA

**DOI:** 10.3201/eid3013.230777

**Published:** 2024-04

**Authors:** Sixtine O. Gurrey, Lara B. Strick, Lana K. Dov, James S. Miller, Monica Pecha, Randy M. Stalter, David L. Miller, Brandon Marshall, Alonso Pezo Salazar, Laura P. Newman

**Affiliations:** Washington State Department of Health, Tumwater, Washington, USA (S.O. Gurrey, L.K. Dov, J.S. Miller, M. Pecha, R.M. Stalter, D.L. Miller, L.P. Newman);; University of Washington, Seattle, Washington, USA (L.B. Strick);; Washington State Department of Corrections, Tumwater (L.B. Strick, B. Marshall, A. Pezo Salazar);; Centers for Disease Control and Prevention, Atlanta, Georgia, USA (R.M. Stalter)

**Keywords:** COVID-19, tuberculosis, respiratory infections, bacteria, severe acute respiratory syndrome coronavirus 2, SARS-CoV-2, SARS, coronavirus disease, zoonoses, viruses, coronavirus, public health practice, prisons, correctional facilities, state government, disease outbreaks, Washington, United States

## Abstract

The large COVID-19 outbreaks in prisons in the Washington (USA) State Department of Corrections (WADOC) system during 2020 highlighted the need for a new public health approach to prevent and control COVID-19 transmission in the system’s 12 facilities. WADOC and the Washington State Department of Health (WADOH) responded by strengthening partnerships through dedicated corrections-focused public health staff, improving cross-agency outbreak response coordination, implementing and developing corrections-specific public health guidance, and establishing collaborative data systems. The preexisting partnerships and trust between WADOC and WADOH, strengthened during the COVID-19 response, laid the foundation for a collaborative response during late 2021 to the largest tuberculosis outbreak in Washington State in the past 20 years. We describe challenges of a multiagency collaboration during 2 outbreak responses, as well as approaches to address those challenges, and share lessons learned for future communicable disease outbreak responses in correctional settings.

The prison environment is often conducive to the spread of various infectious conditions because of factors such as overcrowding, poor ventilation, and increased barriers to accessing timely healthcare (*1*). The higher prevalence of underlying conditions among the incarcerated population compared with the general public puts many persons living in such settings at an increased risk for severe health outcomes (*1*). The large COVID-19 outbreaks in prisons in the Washington (USA) State Department of Corrections (WADOC) system in 2020 highlighted the need for a new public health approach to prevent and control COVID-19 transmission in the system’s 12 facilities and to reduce severe health outcomes. WADOC and the Washington State Department of Health (WADOH) responded to those outbreaks by strengthening partnerships through dedicated corrections-focused public health staff, improving cross-agency outbreak response for coordinating resources (e.g., masks, testing supplies, isolation settings, and staff), implementing and developing corrections-specific public health guidance, and establishing collaborative data systems.

The urgent demands of the COVID-19 response required redirection of limited resources away from other WADOC health services, including tuberculosis (TB) surveillance. In late 2021, staffing shortages, challenges with TB diagnosis, especially amid a concurrent respiratory disease outbreak, and delayed annual TB screenings contributed to a TB outbreak within WADOC, the state’s largest TB outbreak in 20 years. Details about the TB outbreak and Centers for Disease Control and Prevention (CDC) on-site assistance are described elsewhere (*2*). Building on years of partnership and trust between WADOC and WADOH before and during the COVID-19 pandemic, the agencies adapted approaches from the COVID-19 outbreak response to strengthen the TB outbreak response.

To improve responses to future outbreaks and protect the health of persons who are incarcerated and correctional staff, we describe challenges of a multiagency collaboration during an outbreak response, approaches to address those challenges, and share lessons learned. This case study is a companion piece to 2 other articles: a national perspective on lessons learned from the COVID-19 response in correctional and detention facilities (*3*), and a case study on interdisciplinary COVID-19 response in Colorado youth confinement facilities (*4*).

## Strengthening Partnerships through Dedicated Corrections-Focused Public Health Staff

### Challenge

WADOC clinicians and WADOH have long worked together to respond to communicable diseases in Washington state prisons before the COVID-19 pandemic; however, depending on the pathogen, WADOC needed to communicate with the disease-specific WADOH team. Thus, WADOC clinicians needed to identify a new WADOH team with which to work, and a WADOH epidemiologist unfamiliar with correctional settings had to quickly learn about transmission dynamics specific to the prison, prison logistics (e.g., movement tracking), and how to adapt guidance meant for the general public in this complex setting.

### Approach

Given the risk and frequency for COVID-19 outbreaks in correctional facilities, WADOH designated an epidemiologist and clinician to focus on corrections as part of a specialized Outbreak Response in Non-Healthcare Congregate Settings (NHCS) team. This team co-coordinated the COVID-19 outbreak response with WADOC, guided the implementation of outbreak prevention protocols, and helped track case data. During the TB outbreak, the NHCS team used its familiarity with WADOC’s prisons and data systems, and its relationships with WADOC staff, to assist WADOH’s TB team with data collection and contact tracing. That process helped identify >3,000 persons who were considered potentially exposed to someone with contagious TB disease based on time spent in the same airspace, which guided testing recommendations and resource prioritization.

### Lessons Learned

Corrections-focused public health staff can support relationships between agencies during any outbreak by understanding operational challenges in corrections facilities and systems. Those public health staff can assist in developing corrections-specific outbreak-response priorities and feasible control efforts.

## Improving Cross-Agency Outbreak Response Coordination

### Challenge

The complexities of custody operations and staffing limit the feasibility of outbreak control measures in correctional settings. Before the COVID-19 pandemic, WADOH primarily partnered with WADOC clinical leadership, and WADOC custody and operations leadership had limited involvement. During early COVID-19 outbreaks in prisons, that approach created operational barriers in outbreak responses.

### Approach

WADOH and WADOC established weekly COVID-19 coordination meetings beginning in mid-2020 to align cross-agency outbreak response strategies, resources, and communications in a rapidly changing pandemic. In addition to epidemiology and clinical teams, meetings included WADOC emergency operations and custody leadership, which improved coordination between agencies and within WADOC and established a shared understanding of outbreak response approaches and constraints. That coordination also helped with efficient planning and scale-up of new disease prevention processes, including implementation of medical isolation and quarantine areas and expanded COVID-19 testing. That meeting model was adapted for the TB response; WADOC and WADOH weekly meetings similarly assisted in cross-agency communications to address TB-specific operational needs. Those needs included arranging large testing events, determining isolation locations, securing medical equipment and medications, and providing education to staff, residents, and their families.

### Lessons Learned

In large-scale, multifacility outbreaks, frequent coordination meetings with epidemiology, clinical, custody, and operations teams across both agencies helped establish a cohesive response strategy. That coordination enabled WADOC and WADOH to arrange resources for outbreak response, including resources for isolation, testing, and education for staff, residents, and family.

## Following and Developing Corrections-Specific Public Health Guidance

### Challenge

WADOH and WADOC used CDC COVID-19 guidance as a framework for outbreak response in prisons (*5*). Adapting broad recommendations to each prison’s unique operation and built environment and to the rapidly changing knowledge on COVID-19 was challenging. Similar issues with guidance arose when implementing CDC’s recommendations for TB prevention and control in correctional facilities (*6*), including implementing effective infection control and contact tracing, having clear definitions of exposure for airborne pathogens in prisons, managing disincentives for staff and patients to report symptoms, addressing refusals of testing or treatment, determining isolation duration, and identifying alternative isolation spaces when negative pressure rooms were unavailable (*7*).

### Approach

During both outbreaks, WADOH and WADOC, with help from CDC subject matter experts, tailored guidance implementation based on available resources, physical layout, and operational constraints. Solutions included adopting location-based contact tracing and providing incentives for testing, isolation, and treatment in a noncoercive manner rather than by using punishment for persons who refused testing or treatment.

### Lessons Learned

Tailoring disease control guidance toward the needs of a specific correctional system can help to address unique facility needs, educate staff and residents, and prevent the spread of misinformation. Public health and corrections agencies can work together to implement and adapt standard CDC guidance to their needs.

## Establishing Collaborative Data Systems

### Challenge

Because WADOC lacks an electronic medical record (EMR) system, medical data are only accessible by paper charts and non-EMR electronic databases. Reporting of notifiable conditions mainly occurs manually by telephone or fax. During COVID-19 outbreaks, manual case reporting quickly became unmanageable for WADOC, WADOH, and local health jurisdictions. Because TB testing and diagnosis involves tracking multiple clinical results over time, during the TB outbreak, the absence of an EMR system overwhelmed internal data management and created bottlenecks in information sharing with local partners.

### Approach

First, WADOC designated nonclinical staff to collect and manage COVID-19 data electronically. Second, with support from WADOH, WADOC developed technical expertise in database creation and data management to share information electronically across agencies, including case data and test results. Finally, WADOC and WADOH collaborated on new data management and sharing systems. For example, WADOC developed a COVID-19 website dashboard to provide public transparency. The agencies created a shared Research Electronic Data Capture (REDCap) database to track TB exposures in WADOC prisons as new infectious TB cases were identified and as postexposure screening and diagnostic testing were conducted.

### Lessons Learned

Clinical and public health data collection, management, and dissemination require dedicated staff with a shared understanding of public health and corrections agencies’ capabilities and limitations. Securing high-level leadership support and funding to implement modern data systems for both public health and corrections agencies would improve outbreak data surveillance and management.

## Conclusions

The cross-agency, cross-department collaboration and lessons learned from 2 communicable disease outbreak responses in Washington state prisons can serve as a guide to future communicable disease outbreak responses in correctional facilities. Despite the successes of this partnership, institutional barriers and the prison environment itself limited the effect of the efforts of both agencies to reduce the risk for disease transmission. Without an overhaul of the prison system’s physical environment and the criminal legal system, airborne transmission of communicable diseases will continue to be a threat in correctional settings. We highlight the need for sustained resources for public health and corrections partnerships and for tailored communicable disease guidance to support the health of incarcerated persons and correctional staff.
